# Supplementary material for: Aurora B switches relative strength of kinetochore–microtubule attachment modes for error correction
Source: J Cell Biol. 2021 Apr 14;220(6):e202011117. doi: 10.1083/jcb.202011117 (PMC8050843; doi:10.1083/jcb.202011117)
Supplement: Table S1 — lists kinetochore proteins identified in purified KCps by mass spectrometry. [file JCB_202011117_TableS1.docx]

Table S1: Kinetochore proteins identified in purified kinetochore particles by mass spectrometry

| Protein name | Unique peptides | Mol. weight [kDa] | Sequence lengths | Q-value | Score | Sequence coverage | Intensity (iBAQ) |
| --- | --- | --- | --- | --- | --- | --- | --- |
| Ndc80 | 23 | 80.486 | 691 | 0 | 115.53 | 37.3 | 36449000 |
| Spc24 | 11 | 24.603 | 213 | 0 | 87.873 | 70.4 | 97405000 |
| Spc25 | 10 | 25.244 | 221 | 0 | 41.394 | 46.6 | 55587000 |
| Nuf2 | 8 | 52.973 | 451 | 0 | 27.51 | 24.4 | 18298000 |
| Spc105 | 19 | 104.82 | 917 | 0 | 110.14 | 25.8 | 23655000 |
| Kre28 | 8 | 44.674 | 385 | 0 | 56.648 | 26.5 | 40184000 |
| Nnf1 | 11 | 23.639 | 201 | 0 | 107.89 | 50.7 | 610390000 |
| Nsl1 | 17 | 25.416 | 216 | 0 | 207.09 | 77.8 | 472260000 |
| Mtw1 | 26 | 33.243 | 289 | 0 | 187.52 | 58.8 | 418420000 |
| Dsn1 | 9 | 65.691 | 576 | 0 | 37.745 | 22.9 | 10104000 |
| Cnn1 (CENPT) | 1 | 41.312 | 361 | 0 | 8.3498 | 5.5 | 679690 |
| Wip1 (CENPW) | 2 | 10.244 | 89 | 0 | 8.5453 | 39.3 | 1049100 |
| Okp1 (CENPQ) | 14 | 47.349 | 406 | 0 | 89.043 | 38.9 | 29199000 |
| Ame1 (CENPU) | 10 | 37.461 | 324 | 0 | 29.306 | 35.2 | 27016000 |
| Mcm21 (CENPO) | 3 | 42.97 | 368 | 0 | 2.56 | 10.6 | 15056000 |
| Ctf19 (CENPP) | 4 | 42.782 | 369 | 0 | 8.3953 | 13.6 | 7920900 |
| Mif2 (CENPC) | 14 | 62.472 | 549 | 0 | 47.672 | 27 | 40790000 |
| Nkp1 | 7 | 26.976 | 238 | 0 | 30.398 | 34.5 | 39782000 |
| Iml3 (CENPL) | 5 | 28.066 | 245 | 0 | 17.633 | 20.4 | 7922700 |
| Nkp2 | 1 | 17.862 | 153 | 0 | 6.1052 | 7.8 | 4711800 |
| Chl4 (CENPN) | 2 | 52.671 | 458 | 0 | 6.2515 | 7 | 1312700 |
| Mcm16 (CENPH) | 1 | 21.138 | 181 | 0.0016741 | 1.2033 | 5 | 1627800 |
| Mcm22 (CENPK) | 2 | 27.567 | 239 | 0 | 3.0511 | 12.1 | 2709900 |
| H2B2 | 10 | 14.237 | 131;131 | 0 | 64.656 | 64.1 | 733090000 |
| H2A2 | 3 | 13.989 | 132;132 | 0 | 13.501 | 29.5 | 481680000 |
| H4 | 9 | 11.368 | 103 | 0 | 36.809 | 65 | 304380000 |
| Cse4 (CENPA) | 6 | 26.841 | 229 | 0 | 11.637 | 23.1 | 73007000 |
| Skp1 | 8 | 22.33 | 194 | 0 | 28.335 | 45.9 | 89698000 |
| Cep3 (CBF3B) | 1 | 71.357 | 608 | 0.0059653 | 0.91945 | 1.6 | 396870 |
| Mad1 | 6 | 87.65 | 749 | 0 | 16.827 | 12.8 | 2212400 |
| Bub3 | 1 | 38.444 | 341 | 0 | 1.7648 | 5.3 | 745730 |
| Bub1 | 1 | 117.87 | 1021 | 0.010021 | 0.65098 | 1.1 | 0 |
| Fin1 | 3 | 33.186 | 291 | 0 | 8.4003 | 12.7 | 3210200 |
| Stu2 | 2 | 100.92 | 888 | 0 | 28.516 | 3.2 | 583010 |
| Slk19 | 1 | 95.379 | 821 | 0 | 3.3914 | 1.6 | 0 |
| Dam1 | 3 | 38.421 | 343 | 0 | 9.9438 | 13.7 | 18353000 |
| Ask1 | 4 | 32.071 | 292 | 0 | 36.05 | 29.1 | 6556500 |
| Dad3 | 2 | 10.848 | 94 | 0 | 4.6574 | 38.3 | 2579800 |
| Duo1 | 3 | 27.473 | 247 | 0 | 26.239 | 13.4 | 1215200 |
| Dad2 | 1 | 15.071 | 133 | 0 | 1.8749 | 8.3 | 0 |
| Dad4 | 1 | 8.1552 | 72 | 0 | 2.8758 | 20.8 | 0 |
| Hsk3 | 1 | 8.0881 | 69 | 0 | 7.3375 | 15.9 | 0 |
| Spc19 | 0 | 18.9007 | 165 | - | - | - | 0 |
| Spc34 | 0 | 34.0802 | 295 | - | - | - | 0 |
| Dad1 | 0 | 10.5067 | 94 | - | - | - | 0 |

Unique peptides: Number of Unique peptides identified for each protein

Molecular weight: molecular weight of the protein in kilo daltons

Sequence lengths: The amino acid length of the protein

Q-Value: a measure of false discovery rates. (Stringency of FDR (false discovery rates) cut-off)

Score: Highest Andromeda score

Sequence coverage: The % of total sequence of the protein covered by the identified peptides.

iBAQ: Intensity-based absolute quantification.

The proteins in the same sub-complex are highlighted in the same colour.
